# Characterization of *trans*-spliced chimeric RNAs: insights into the mechanism of *trans*-splicing

**DOI:** 10.1093/nargab/lqae067

**Published:** 2024-06-06

**Authors:** Rui Yokomori, Takehiro G Kusakabe, Kenta Nakai

**Affiliations:** Human Genome Center, Institute of Medical Science, The University of Tokyo, Tokyo 108-8639, Japan; Institute for Integrative Neurobiology, Graduate School of Natural Science, Konan University, Kobe 658-8501, Japan; Department of Biology, Faculty of Science and Engineering, Konan University, Kobe 658-8501, Japan; Human Genome Center, Institute of Medical Science, The University of Tokyo, Tokyo 108-8639, Japan

## Abstract

*Trans*-splicing is a post-transcriptional processing event that joins exons from separate RNAs to produce a chimeric RNA. However, the detailed mechanism of *trans*-splicing remains poorly understood. Here, we characterize *trans*-spliced genes and provide insights into the mechanism of *trans*-splicing in the tunicate *Ciona*. Tunicates are the closest invertebrates to humans, and their genes frequently undergo *trans*-splicing. Our analysis revealed that, in genes that give rise to both *trans*-spliced and non-*trans*-spliced messenger RNAs, *trans*-splice acceptor sites were preferentially located at the first functional acceptor site, and their paired donor sites were weak in both *Ciona* and humans. Additionally, we found that *Ciona trans*-spliced genes had GU- and AU-rich 5′ transcribed regions. Our data and findings not only are useful for *Ciona* research community, but may also aid in a better understanding of the *trans*-splicing mechanism, potentially advancing the development of gene therapy based on *trans*-splicing.

## Introduction

*Trans*-splicing is a post-transcriptional event in which exons from two separate RNAs are joined to produce a chimeric RNA. Since the first discovery in trypanosomes (1,2), *trans*-splicing has been reported in various species from lower eukaryotes to vertebrates (3). While the fraction of genes that undergo *trans*-splicing varies across species, *trans*-splicing is particularly common in the nematode *Caenorhabditis elegans* and the tunicate *Ciona* (4).

The *trans*-splicing in *C. elegans* and *Ciona* is known as spliced-leader (SL) *trans*-splicing, in which the 5′ exon of a small noncoding RNA, called SL RNA, is spliced to the *trans*-splice acceptor site of a pre-messenger RNA (pre-mRNA). The 5′ region upstream of the *trans*-splice acceptor site, called outron, is discarded during *trans*-splicing. In the tunicate *Ciona intestinalis* type A (*Ciona robusta*), ∼50% of genes are thought to undergo SL *trans*-splicing, in which the 16-nt 5′ exon of a 46-nt SL RNA is joined to the *trans*-splice acceptor site of a pre-mRNA (4–6). Although the function of chimeric RNAs produced by SL *trans*-splicing is not yet fully understood, a previous analysis has shown that *trans*-spliced chimeric RNAs in *C. elegans* have higher translational efficiency than non-*trans*-spliced RNAs transcribed from the same gene (7).

In vertebrates, *trans*-splicing is a rare event. The SL *trans*-splicing has not yet been observed (8), and to our knowledge, no reports have provided evidence of a common *trans*-splicing mechanism shared between vertebrates and invertebrates. In humans, chimeric RNAs are thought to be generated solely by chromosomal rearrangements in cancer cells (9). However, comprehensive analysis of massive RNA sequencing (RNA-seq) libraries reported many (∼300) possible recurrent chimeric RNAs in normal adult tissues and cells, which do not have chromosomal rearrangements, suggesting that they are produced by an RNA-level event, such as *trans*-splicing (9,10). While only a handful of these chimeric RNAs are experimentally confirmed, a previous extensive computational analysis has detected an unexpectedly large number of chimeric RNAs that might result from *trans*-splicing (9–11). Some studies of human embryonic stem cells (ESCs) showed that the long noncoding RNA *RMST* is intragenically *trans*-spliced, and resulting chimeric RNAs contribute to pluripotency maintenance of ESCs by repressing differentiation-related transcription factors and WNT signaling pathway through the PRC2 complex and NANOG (12,13).

Although the mechanism of *trans*-splicing is not yet fully understood, its principle has been applied to gene therapy for human genetic disorders, such as Duchenne muscular dystrophy, epidermolysis bullosa simplex and Huntington’s disease (8,14–16). In *trans*-splicing-based gene therapy, an exogenous artificial RNA, called pre-mRNA *trans*-splicing molecule, is used to replace mutated exons of an endogenous pre-mRNA transcribed from causal genes. By properly designing the *trans*-splicing molecule, the replacement of mutated exons can occur at 5′ exons, 3′ exons and even internal exons. However, the replacement rate (i.e. the efficiency of *trans*-splicing) is still low to obtain a significant therapeutic effect due to poor understanding of the *trans*-splicing mechanism (8,17).

Here, we aim to characterize *trans*-spliced genes and provide new insights into the *trans*-splicing mechanism in *Ciona*, a species of tunicates, which are the closest invertebrates to humans (18). We first re-assemble transcripts using a large set of public RNA-seq data to detect 5′ transcribed regions upstream of *trans*-splice acceptor sites (i.e. outrons) that are not annotated in the current model (the KY2019 model) due to efficient *trans*-splicing in *Ciona* (19,20). We then characterize *trans*-spliced genes against non-*trans*-spliced genes in terms of 5′ transcribed regions and splice sites. We also aim to analyze putative human *trans*-spliced genes and compare their characteristics between *Ciona* and humans.

## Materials and methods

### Re-assembling *Ciona* transcripts

Transcripts were re-assembled using 82 RNA-seq samples (Supplementary Table S1). The RNA-seq reads were preprocessed using cutadapt v1.11 (21) (Supplementary Table S2). The preprocessed reads were mapped to the Hoya T-strain (HT) genome (14 chromosomes only) (19) using STAR v2.7.9a (22). Transcripts were assembled using StringTie v1.2.3 (23) and Scallop v0.10.4 (24). Additional information can be found in Supplementary Methods.

### Identification of *trans*-splice acceptor sites and transcription start sites

*Ciona trans*-splice acceptor sites and transcription start sites (TSSs) were identified using our previously published TSS-seq data (25). TSS-seq is a method for precisely identifying TSSs at a genome-wide level (26). It employs an oligo-capping method (27), which specifically replaces the 5′ cap structure of mRNA with a synthetic oligoribonucleotide to label the 5′ ends of mRNAs. This method, combined with next-generation sequencing, allows us to obtain reads that represent the 5′ end sequences of mRNAs. Here, we classified *Ciona* TSS-seq reads into two categories: those from SL *trans*-spliced RNAs and those from non-*trans*-spliced RNAs based on the presence or absence of the 5′ SL sequence. These categories were then used to identify *trans*-splice acceptor sites and TSSs, respectively, by mapping the reads to the HT genome using STAR v2.7.9a (22). Because we have previously shown that the TSS-seq data included artifacts not representing real TSSs (25), open chromatin regions identified using ATAC-seq data (28) were also used as supportive evidence of true TSSs. Specifically, we first identified TSS clusters within the regions around 5′ ends of transcripts or upstream of *trans*-splice acceptor sites, and then determined putative TSSs based on those overlapping with open chromatin regions. For human *trans*-spliced genes, TSSs were manually determined based on TSS clusters at promoters previously identified using TSS-seq data (25). Human *trans*-splice acceptor sites were retrieved from published papers (10,13,29,30). Additional information can be found in Supplementary Methods.

### Local enrichment analysis of nucleotide content

The local enrichment of nucleotide content was examined using a 30-bp sliding window. For each window, the nucleotide content was tested to determine whether it is higher in *trans*-spliced genes than in non-*trans*-spliced genes. *P*-values were calculated using one-sided Mann–Whitney *U* test and adjusted using false discovery rate (FDR) correction (31).

### Local motif enrichment analysis

Motifs were first evaluated whether they show the enrichment in target (*trans*-spliced gene) sequences compared to background sequences using the binomial test:


\begin{equation*}P = \left( {\begin{array}{@{}*{1}{c}@{}} n\\ k \end{array}} \right){{p}^k}{{\left( {1 - p} \right)}^{n - k}},\end{equation*}

where *n*, *k* and *p* represent the total number of target sequences, the number of target sequences with the motif in a window and the percentage of background sequences with the motif in the same window, respectively. The background sequences were randomly generated from intergenic regions (*n* = 3000). *P*-values were calculated for all windows and adjusted using FDR correction. The windows with FDR < 0.05 were considered candidates of locally enriched regions. The motifs were next evaluated whether they show the enrichment in the candidate windows of target sequences compared to the same windows of control (non-*trans*-spliced gene) sequences using the Fisher’s exact test:


\begin{equation*}P = \frac{{\left( {\begin{array}{@{}*{1}{c}@{}} C\\ x \end{array}} \right)\left( {\begin{array}{@{}*{1}{c}@{}} {N - C}\\ {R - x} \end{array}} \right)}}{{\left( {\begin{array}{@{}*{1}{c}@{}} N\\ R \end{array}} \right)}},\end{equation*}

where *N*, *C*, *R* and *x* represent the total number of target and control sequences, the total number of target and control sequences with the motif in a window, the number of target sequences and the number of target sequences with the motif in the window, respectively. The motifs with at least one significant window (FDR < 0.05) were considered locally enriched motifs. Motif binding sites were predicted using FIMO v5.0.1 (32). Additional information can be found in Supplementary Methods.

## Results

### Re-assembling *Ciona* transcripts

To identify the 5′ transcribed regions upstream of *trans*-splice acceptor sites, *Ciona* transcriptome was re-assembled in the HT genome using 82 RNA-seq samples. This analysis led us to discover 7735 novel transcripts that had an extended 5′ exon or novel exons upstream of known transcripts. These regions may represent either complete or partial outrons. Indeed, we confirmed the presence of an outron in a novel transcript for troponin I gene (KY2019:KY.Chr11.476), which is one of the first *Ciona* genes confirmed to be *trans*-spliced and is the only gene whose TSS has been experimentally validated before (5,33) (Supplementary Figure S1). For our analysis, we combined these newly identified transcripts with the annotated transcripts from the KY2019 core model (19). The gene bodies of known gene loci were redefined based on the combined transcript set. Since we only added newly discovered transcripts associated with known gene loci, the total count of genes (13 801 genes) on the 14 chromosomes remained unchanged. These genes included 1404 operonic and 12 397 non-operonic genes. The operonic genes were identified using our in-house script because the KY2019 model does not provide the annotation of operons. In our script, operonic genes were defined as genes from putative polycistronic regions where two or more genes are present consecutively on the same strand with no intergenic distance (6), and where the 5′ ends of all the genes except the first gene are annotated as *trans*-splice acceptor sites (34). The number of operonic genes in the HT genome was smaller than that (*n* = 2909) in the previous Kyoto Hoya (KH) genome assembly (35). This is, at least in part, due to the difference of the definition of intergenic distances; putative polycistronic regions whose intergenic distances were <100 bp were defined as operons in the previous KH annotation (35).

### Identifying *trans*-spliced genes

To compare with human genes, we focused on non-operonic *Ciona* genes (*n* = 12 397). These genes were subsequently classified into *trans*-spliced and non-*trans*-spliced genes based on the presence or absence of *trans*-splice acceptor sites on their redefined gene body. We used *trans*-splice acceptor sites from two different sources: (i) *trans*-splice acceptor sites (*n* = 56 126) identified in this study using genome-wide high-throughput TSS-seq data (Supplementary Methods) and (ii) those (*n* = 15 512) annotated in the KY2019 model primarily identified using expressed sequence tags (ESTs), including 5′ full-length ESTs and high-throughput SL mRNA-derived reads (19,20). The set of TSS-seq-based *trans*-splice acceptor sites was much larger than the KY2019-based set and was therefore used as the primary dataset.

In *Ciona*, we identified 9850 *trans*-spliced genes with TSS-seq-based *trans*-splice acceptor sites as well as 2535 non-*trans*-spliced genes with neither TSS-seq-based nor KY2019-based *trans*-splice acceptor sites. The remaining 12 genes were considered ambiguous genes because they did not have TSS-seq-based but KY2019-based *trans*-splice acceptor sites. The *trans*-spliced genes had, on average, three to four TSS-seq-based *trans*-splice acceptor sites (Supplementary Figure S2). The most frequently used *trans*-splice acceptor site among these was defined as the major *trans*-splice acceptor site. Many minor *trans*-splice acceptor sites were closely distributed within 50 bp of major sites, consistent with a previous study (20) (Supplementary Figure S3). Of the 9850 *trans*-spliced genes, 6640 (67%) had KY2019-based annotated *trans*-splice acceptor sites, and their major *trans*-splice acceptor sites were located at the annotated sites in 6005 (90%) of them. The remaining 3210 (33%) lacked KY2019-based annotated *trans*-splice acceptor sites. In humans, we identified 52 *trans*-spliced genes including the previously reported *RMST* gene as well as 24 808 non-*trans*-spliced genes using published data (10,13).

### *Trans*-splicing preferentially occurs at the first functional acceptor site

We first examined the location of *trans*-splice acceptor sites on *trans*-spliced genes. Since *Ciona trans*-spliced genes had multiple TSS-seq-based *trans*-splice acceptor sites, the location was determined according to their major *trans*-splice acceptor site (see Supplementary Methods). Unless otherwise specified, *trans*-splice acceptor sites of *trans*-spliced genes represent major *trans*-splice acceptor sites.

**Figure 1.**
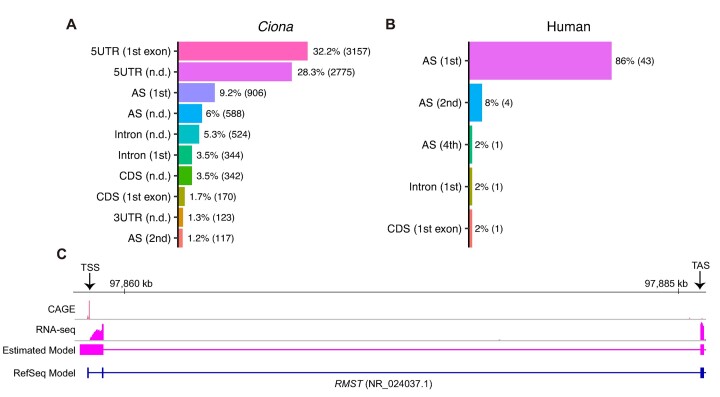
Locations of *trans*-splice acceptor sites. (**A**, **B**) Top 10 locations of *trans*-splice acceptor sites in *Ciona* (*n* = 9817) and humans (*n* = 50). Each bar represents the percentage of major *trans*-splice acceptor sites at a given location relative to the total number of major *trans*-splice acceptor sites. The number in parentheses represents the number of major *trans*-splice acceptor sites. The location was determined based on the newly discovered transcripts and KY2019 transcripts (19). KY2019 comprises three types of transcripts (non-SL, SL and ND) depending on their 5′ ends, representing TSSs, *trans*-splice acceptor sites and undetermined status, respectively. The description in parentheses on the left side of each feature shows which feature the *trans*-splice acceptor site was located on. The ordinal number and ‘1st exon’ indicate that it was on the *N*th feature and the feature on the first exon of newly discovered or non-SL transcripts, respectively, while ‘n.d.’ (not determined) indicates that it is on the feature of SL or ND transcripts. AS, acceptor site. (**C**) Gene tracks showing the TSS and *trans*-splice acceptor site of *RMST* gene. The first track represents cap-analysis gene expression (CAGE) TSSs identified using FANTOM5 human CAGE libraries (*n* = 1897) (47). The novel transcript estimated using a human ESC RNA-seq sample was shown in the third track. The CAGE data exhibited not only the strongest peak, but also a weak peak ∼65 bp upstream of the strongest peak. These strongest and weak peaks were considered to represent the major TSSs of the estimated and RefSeq transcripts, respectively. The 5′ end of the estimated model was upstream of the TSS peaks, suggesting the presence of very minor TSSs. TAS, *trans*-splice acceptor site.

We obtained 9817 and 50 *trans*-splice acceptor sites from *Ciona* and human *trans*-spliced genes, respectively. The number of *trans*-splice acceptor sites was slightly fewer than the number of *trans*-spliced genes due to some overlapping genes in the genome. In *Ciona*, ∼60% of *trans*-splice acceptor sites were located in the 5′ untranslated region (UTR), and at least half of these (32% of the total) were in the gene’s first exon (Figure 1A). Unfortunately, we could not determine whether all of the *trans*-splice acceptor sites in the 5′ UTR were in the first exon due to transcripts with undetermined 5′ ends. *Trans*-splice acceptor sites were also frequently found at the first *cis*-splice acceptor site (Figure 1A). In humans, while *trans*-splice acceptor sites were not found in the 5′ UTR of the first exon, they were predominantly located at the first *cis*-splice acceptor site (Figure 1B). We also confirmed that the *trans*-splice acceptor site of *RMST* gene was located at the first acceptor site using RNA-seq data showing that an alternative isoform different from the RefSeq transcript (NR_024037.1) was expressed in ESCs (Figure 1C). The observed high *trans*-splicing frequency at the first *cis*-splice acceptor site was not obtained by chance in either *Ciona* or humans, as the proportion of the first *cis*-splice acceptor sites undergoing *trans*-splicing was significantly higher compared to the background proportion (*P* < 0.01, binomial test; Supplementary Figure S4).

Overall, our results indicated that *trans*-splicing preferentially occurred at the first functional acceptor site in both *Ciona* and humans. We therefore focused on two groups of *trans*-spliced genes for further analysis: those with major *trans*-splice acceptor sites in the 5′ UTR of the first exon and those with major *trans*-splice acceptor sites at the first *cis*-splice acceptor site (1st AS), which we refer to as TS-5UTR and TS-1stAS, respectively. In addition, to compare splice sites between *trans*-spliced and non-*trans*-spliced genes, we focused on 1631 and 23 745 non-*trans*-spliced genes with *cis*-splice sites for further analysis in *Ciona* and humans, respectively.

### Identifying TSSs

We next aimed to identify TSSs using TSS-seq and ATAC-seq data to determine the accurate 5′ transcribed region, the first exon or the first splice site position for each gene. As a result, we successfully identified representative (or major) TSSs for 392 TS-1stAS genes, 515 TS-5UTR genes and 157 non-*trans*-spliced genes in *Ciona* (Supplementary Table S3; Supplementary Methods). TSS identification failed for 506 TS-1stAS genes, 2584 TS-5UTR genes and 1421 non-*trans*-spliced genes. Additionally, we excluded 8 TS-1stAS genes, 63 TS-5UTR genes and 53 non-*trans*-spliced genes mainly because they could not be reliably classified as *trans*-spliced or non-*trans*-spliced genes due to very short outrons or other reasons (Supplementary Methods). Of the 907 (392 + 515) *trans*-spliced genes for which putative TSSs were successfully identified, 477 (53%) did not have KY2019-based annotated TSSs previously determined using 5′ full-length ESTs of non-*trans*-spliced RNAs (19,35). The putative TSSs of the remaining 430 (47%) *trans*-spliced genes were, on average, located 154 bp upstream of their annotated TSSs (Supplementary Figure S5), suggesting that they represent alternative upstream promoters of these genes. In contrast, the identified putative TSSs of non-*trans*-spliced genes were mostly located within 15 bp from their annotated TSSs (Supplementary Figure S5). For human dataset, we identified TSSs of 34 human *trans*-spliced genes (TS-1stAS genes) and 5674 non-*trans*-spliced genes using TSS-seq data (Supplementary Table S4).

### *Trans*-spliced genes have the weaker first donor site than non-*trans*-spliced genes

**Figure 2.**
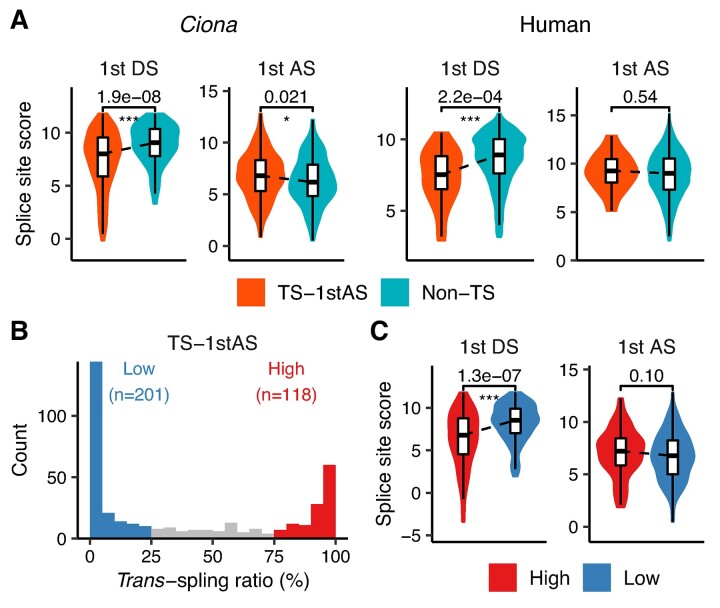
Association between splice site strength and *trans*-splicing. (**A**) Violin plots showing the scores of *trans*-spliced (TS-1stAS) and non-*trans*-spliced (non-TS) genes. (**B**) Histogram showing the distribution of *trans*-splicing ratio for TS-1stAS genes. The number of TS^High^ and TS^Low^ genes is shown. (**C**) Violin plots showing the splice site scores of TS^High^ and TS^Low^ genes. In panels (A) and (C), outliers, which fall below the first quartile − 1.5 × interquartile range (IQR) or above the third quartile + 1.5 × IQR, were removed. Two-sided Mann–Whitney *U* test was used for statistical comparison. *P*-values from the 1st DS and AS score comparisons were adjusted using FDR correction (the Benjamini–Hochberg procedure) (31)., FDRs are shown on the violin plots. ***, FDR < 0.001; **, FDR < 0.01; *, FDR < 0.05; DS, donor site; AS, acceptor site.

Considering the prevalence of *trans*-splice acceptor sites at the first *cis*-splice acceptor site (Figure 1A and B), we investigated whether the strength of the first splice site is different between *trans*-spliced (TS-1stAS) and non-*trans*-spliced genes. We estimated the splice site strength using the maximum entropy model (MaxEnt) (36), a splice site model that assigns a score to a 9-bp donor site sequence (−3 to +6 from a donor site) or a 23-bp acceptor site sequence (−20 to +3 from an acceptor site). The MaxEnt score represents the strength or confidence of a given sequence being a true splice site; the higher the score, the more likely the sequence is a splice site (stronger splice site), while the lower the score, the less likely it is a splice site (weaker splice site) (37). We found that the first donor sites of *trans*-spliced (TS-1stAS) genes showed significantly lower scores than those of non-*trans*-spliced genes in both *Ciona* and humans (Figure 2A; FDR < 0.001; two-sided Mann–Whitney *U* test), suggesting that *trans*-spliced genes have the weaker first donor sites than non-*trans*-spliced genes. On the other hand, the first acceptor sites tended to have higher scores for *trans*-spliced genes, while they did not show as statistically significant differences as observed in the first donor sites (Figure 2A).

### The first donor site strength is associated with *trans*-splicing efficiency

Given that the first donor sites were weaker in *trans*-spliced genes, we further examined whether their strength is associated with *trans*-splicing efficiency. To this end, *Ciona trans*-spliced (TS-1stAS) genes were classified into TS^High^ and TS^Low^ groups according to *trans*-splicing ratio, which was estimated by the number of TSS-seq tags mapped to the TSS and *trans*-splice acceptor site corresponding to the expression level of non-*trans*-spliced and *trans*-spliced RNAs, respectively (Figure 2B). The comparison between the two groups revealed that the donor site scores were significantly lower in the TS^High^ group than in the TS^Low^ group (Figure 2C; FDR < 0.001; two-sided Mann–Whitney *U* test), suggesting that the weaker first donor sites are associated with higher *trans*-splicing efficiency. This significant difference was observed even when different thresholds for *trans*-splicing ratio were used to define the two groups (Supplementary Figure S6). Additionally, the first acceptor sites tended to have higher scores in the TS^High^ group (Supplementary Figure S6).

### The 5′ transcribed regions upstream of the first acceptor sites have higher G + U and A + U contents in *Ciona*

We next investigated nucleotide content (*N* content and *N* + *N* content) of the 5′ transcribed regions upstream of the first acceptor sites (1st ASs), which correspond to the first exon and intron. These regions are specifically called outrons for *trans*-spliced genes in *Ciona*. Their median lengths were 458 and 453 bp for *trans*-spliced (TS-1stAS) and non-*trans*-spliced genes, respectively, in *Ciona* (Supplementary Figure S7A). In humans, these lengths were 7622 and 3098 bp, respectively (Supplementary Figure S7B).

**Figure 3.**
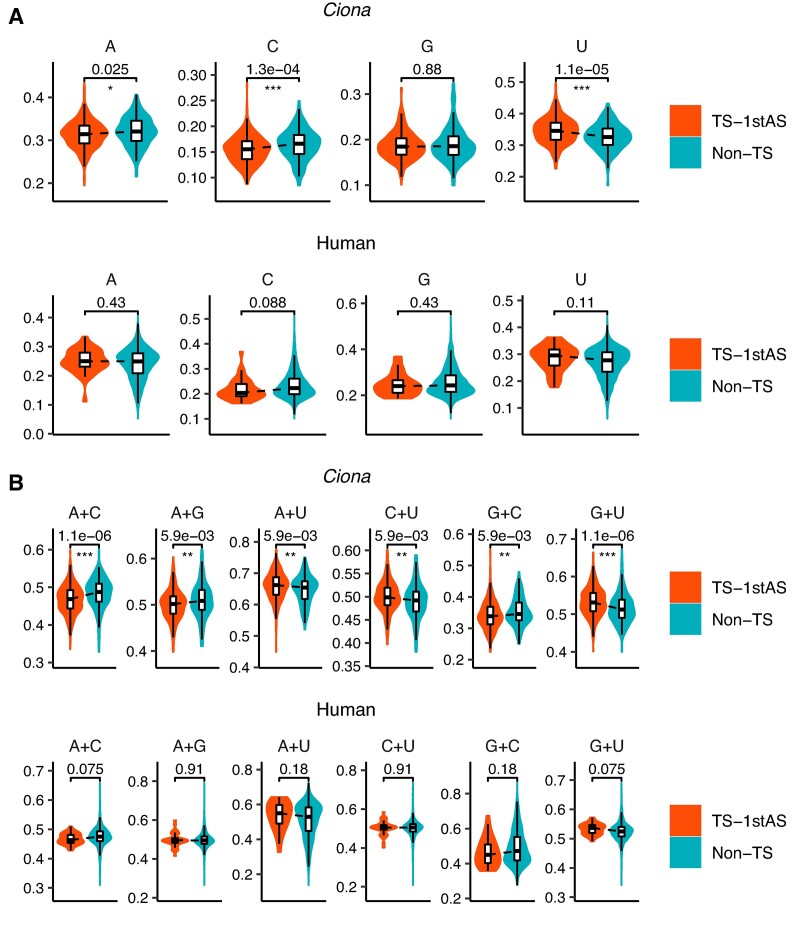
Violin plots showing the nucleotide content of the first exon and intron regions in *trans*-spliced (TS-1stAS) and non-*trans*-spliced (non-TS) genes. Two-sided Mann–Whitney *U* test was performed for each nucleotide content. *P*-values from four (**A**) or six (**B**) different nucleotide content comparisons were adjusted using FDR correction (the Benjamini–Hochberg procedure) (31). FDRs are shown on the violin plots. ***, FDR < 0.001; **, FDR < 0.01; *, FDR < 0.05.

In *Ciona*, *trans*-spliced genes had significantly higher U content than non-*trans*-spliced genes in the first exon and intron regions (i.e. outrons) (Figure 3A). This higher U content was also observed in the exon and intron regions downstream of the first acceptor sites (Supplementary Figures S8A and S9A), suggesting that the higher U content itself may not be the sole determinant of frequent *trans*-splicing at the first acceptor site. Interestingly, the first exon and intron regions showed higher U content in human *trans*-spliced genes as well, although statistical significance was not observed (Figure 3A). Additionally, they showed a pattern of nucleotide content differences similar to that observed in *Ciona* (Supplementary Figure S10A).

The *N* + *N* content analysis in *Ciona* revealed that the first exon and intron regions, or outrons, had significantly higher G + U, A + U and C + U contents in *trans*-spliced genes than in non-*trans*-spliced genes (Figure 3B and Supplementary Figures S8B and S9B). Among these three different contents, only G + U content showed a larger fold change difference in the first exon and intron regions compared to the exon and intron regions downstream of the first acceptor sites (Supplementary Figure S10B), suggesting that the higher G + U content is more associated with outrons.

In humans, *trans*-spliced genes showed higher G + U and A + U contents in the first exon and intron regions, although statistical difference was not observed (Figure 3B). Similarly, we did not observe statistically significant differences in the exon and intron regions downstream of the first acceptor sites (Supplementary Figures S8B and S9B). Unlike *Ciona*, A + U content showed a larger fold change difference in the first exon and intron regions compared to the downstream exon and intron regions in humans (Supplementary Figure S10B).

### G + U and A + U contents are locally enriched in the 5′ transcribed regions of *Ciona trans*-spliced genes

**Figure 4.**
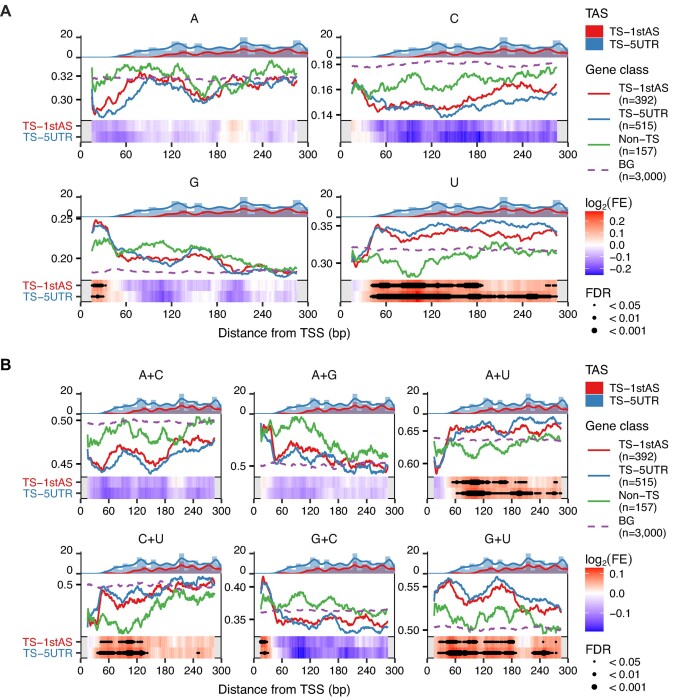
Local enrichment analysis of *N* content (**A**) and *N* + *N* content (**B**) in 5′ transcribed regions. The line graph shows nucleotide content calculated using a 30-bp sliding window. The heatmap on the bottom shows the fold enrichment (FE) of nucleotide content in the window of TS-1stAS or TS-5UTR genes relative to non-*trans*-spliced (non-TS) genes. The red and blue histograms on the top show the frequency of *trans*-splice acceptor sites (TASs) in TS-1stAS and TS-5UTR genes, respectively, with the density plot of the TAS frequency overlaying the histograms. Points on the heatmap represent the statistical significance (FDR) level of enrichment in each window. Background sequences (BGs) were generated by randomly extracting sequences from intergenic regions. The number of genes in each group is shown in parentheses.

We further analyzed the local enrichment of nucleotide content within the 5′ transcribed regions in *Ciona*. *Trans*-spliced genes (TS-1stAS and TS-5UTR) exhibited significantly higher U content and A + U content than non-*trans*-spliced genes (Figure 4A and B). In addition, as shown in the top histogram and density plots in Figure 4, we observed the presence of *trans*-splice acceptor sites whose flanking regions have been previously shown to be AU-rich (20), raising the possibility that the observed enrichment simply reflects the AU-rich regions. However, the enriched U and A + U contents were consistently observed regardless of the outron length of the *trans*-spliced genes (Supplementary Figure S11A and B), suggesting that they are not solely due to AU richness around *trans*-splice acceptor sites. Moreover, *trans*-spliced genes showed higher G content near TSSs than non-*trans*-spliced genes (Figure 4A). The analysis of *N* + *N* content revealed that *trans*-spliced genes had elevated levels of G + U content compared to non-*trans*-spliced genes (Figure 4B). These enrichments were also consistently observed regardless of their outron length (Supplementary Figure S11A and B). Additionally, *trans*-spliced genes exhibited significantly higher A + U content around the first acceptor sites than non-*trans*-spliced genes (Supplementary Figures S12 and S13).

### Local enrichment analysis of known RBP motifs

**Figure 5.**
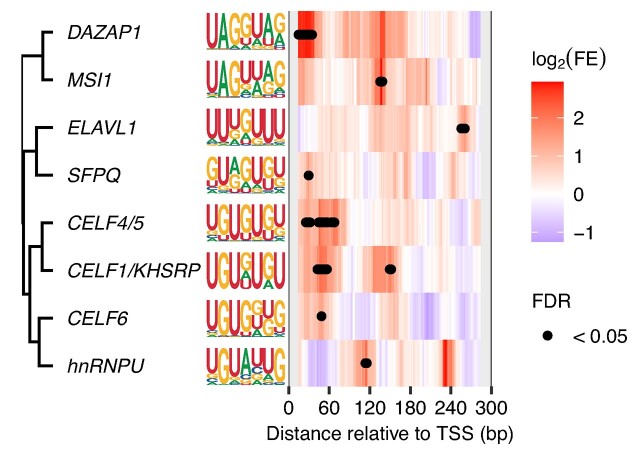
Local enrichment of known RBP motifs. The heatmap shows the fold enrichment (FE) of the motifs in each window (30 bp) of *trans*-spliced genes relative to non-*trans*-spliced genes. The dendrogram on the left was generated using the complete linkage method applied to the dissimilarity distance matrix. The dissimilarity between motifs was calculated using the Euclidean distance between vectors of the log_2_(FE) values of the windows.

Taking into account the local enrichment of nucleotide contents, we investigated whether known human RNA-binding protein (RBP) motifs (157 nonredundant motifs) were locally enriched in the 5′ transcribed regions of *Ciona trans*-spliced genes. Human RBP motifs were obtained from the ATtRACT database and a previous study (38,39), and redundancy within the motifs was eliminated by merging similar motifs (Supplementary Methods). Out of the motifs analyzed, eight motifs exhibited statistically significant enrichment (Figure 5). In particular, deleted in azoospermia-associated protein 1 (DAZAP1), a member of the hnRNP family, showed the strongest enrichment in the 5′ transcribed regions near TSSs. On the other hand, none of RBP motifs showed significant enrichment around the first acceptor sties. The *Ciona* homologs of the eight human RBPs were found using BLAST+ (40) (Supplementary Table S5).

## Discussion

*Trans*-splicing is a common event in diverse invertebrate species such as *C. elegans* and *Ciona*. Although it is rare in humans, its principle has been utilized for gene therapy. However, poor knowledge of *trans*-splicing mechanism hinders the improvement of its accuracy and efficiency. In this study, we found several features that may be associated with *trans*-splicing control in *Ciona* and humans. To the best of our knowledge, this is the first study to identify putative TSSs for over 900 *Ciona trans*-spliced genes and analyze their 5′ transcribed regions and splice sites.

The *trans*-splice acceptor sites of *trans*-spliced genes were preferentially located at the first functional acceptor site in both *Ciona* and humans. While it was previously thought that only pre-mRNAs without a donor site upstream of the *trans*-splice acceptor site could undergo *trans*-splicing (41), our result suggests that *trans*-splicing can occur at the first acceptor site with or without its paired upstream donor site. Importantly, *trans*-spliced (TS-1stAS) genes had the weak first donor sites associated with high *trans*-splicing ratio in *Ciona*. A previous study on gene therapy approach in human cells has shown that 3′ *trans*-splicing, which replaces mutated 3′ exons with normal exons, successfully occurred for the target intron with the weaker 3′ splice site (42). Taken together, our results suggest that the strength of splice donor sites upstream of *trans*-splice acceptor sites is a critical factor that determines the efficiency of *trans*-splicing in both *Ciona* and humans.

Our discovery, linking the strength of splice donor sites to *trans*-splicing efficiency, suggests the plausibility of a single-promoter hypothesis for SL *trans*-splicing (20), in which both *trans*-spliced (TS-1stAS) and non-*trans*-spliced RNAs originate from the same promoter located upstream of the first donor site. In this model, *trans*-spliced (TS-1stAS) RNAs can be competitively produced alongside non-*trans*-spliced ones transcribed from the same promoter, with variable *trans*-splicing efficiency depending, at least in part, on the strength of the first donor sites upstream of *trans*-splice acceptor sites (the first acceptor sites).

However, our finding does not rule out a two-promoter hypothesis, in which *trans*-spliced and non-*trans*-spliced RNAs are transcribed from separate promoters, respectively (20). For example, as illustrated in (20), it is plausible that *trans*-spliced (TS-1stAS) RNAs originate from an alternative promoter within the first intron (i.e. between the first donor and acceptor sites) regardless of the first donor site strength. It is also plausible that non-*trans*-spliced RNAs are transcribed from a promoter upstream of and close to the *trans*-splice acceptor site, while *trans*-spliced (TS-5UTR) RNAs are transcribed from a separate promoter further upstream, with sufficient length of outrons (20). To validate the single-promoter hypothesis, a genetic experiment assessing the abundance of *trans*-spliced RNAs when their promoter is deleted or blocked is essential.

In *Ciona*, the outrons of *trans*-spliced genes displayed high A + U content. This is consistent with a previous *C. elegans* study that has shown that AU richness in outrons affects *trans*-splicing efficiency (43). Furthermore, *Ciona trans*-spliced genes exhibited higher G + U content in their 5′ transcribed regions near TSSs than non-*trans*-spliced genes, consistent with our previous study (25). These nucleotide enrichments may result from binding sites of RBPs. Interestingly, a recent study has suggested that a homologous protein of human TIA1, which binds to U-rich sequences, is associated with *trans*-splicing in *C. elegans* (44). Our local enrichment analysis of human RBP motifs revealed that several splicing factors such as DAZAP1 were enriched in the 5′ transcribed regions. Although the function of predicted binding sites is unknown, our results suggest that known or unknown splicing factors binding to GU/AU-rich sites may play a role in *trans*-splicing control. Additionally, the enrichment of the specific nucleotides might result from elements hybridizing with the SL RNA. A previous study in *C. elegans* has shown that *trans*-splicing of polycistronic pre-mRNAs requires a U-rich element in intercistronic regions, which hybridizes with the SL RNA (45). Interestingly, *Ciona* SL RNA possesses a functionally unknown AU/CA-rich region at its 3′ region, which could potentially hybridize with AU/GU-rich elements (Supplementary Figure S14), although experimental evidence is currently lacking.

It is important to note that our study only characterized a small subset of two gene groups: *trans*-spliced genes and non-*trans*-spliced but *cis*-spliced genes, representing ∼9 percent (907 of 9850) and 10 percent (157 of 1631), respectively, of the estimated total *in Ciona*. The limited total number of genes analyzed and the difference in their percentages between gene groups may potentially affect the results. While our approach in this study, using the combination of TSS-seq and ATAC-seq, enabled us to increase the total number of non-operon gene TSSs analyzed (1064 genes) compared to our previous study (610 genes), which only used the same TSS-seq dataset (25), more comprehensive analysis using larger-scale next-generation sequencing data is necessary in future. Additionally, while operon genes were excluded from the analysis for comparison with human genes, characterizing operon *trans*-splicing is also a future task. Furthermore, TSSs identified in this study are putative and were not determined through experimental methods directly associating them with individual genes. The TSSs for each gene group therefore may potentially include incorrect TSSs. Moreover, since our local enrichment analysis is based on computational prediction, it is important to validate whether the predicted binding sites of candidate RBPs and the 3′ region of the SL RNA are involved in *trans*-splicing control or not. Further functional experiment is also required to elucidate proteins binding to *trans*-spliced RNAs. Lastly, due to the very limited number of human *trans*-spliced genes, we were not able to conduct nucleotide enrichment analysis in humans similar to what we did in *Ciona*. It remains unknown whether human *trans*-spliced genes exhibit the enrichment of specific nucleotides.

In conclusion, this study reported several characteristics that may be associated with *trans*-splicing control in terms of 5′ transcribed regions and splice sites. Interestingly, some characteristics were conserved between *Ciona* and humans. Our findings will not only help us better understand *trans*-splicing mechanism, but also have the potential to contribute to the development of more efficient *trans*-splicing-based gene therapy.

## Supplementary Material

lqae067_Supplemental_Files

## Data Availability

The TSS-seq data for *Ciona* TSSs and *trans*-splice acceptor sites are available in the NCBI Sequence Read Archive (SRA) with accession number SRP063032. The *Ciona* ATAC-seq data are available in the NCBI Gene Expression Omnibus with accession number GSE126691. The SRA accession numbers for the *Ciona* RNA-seq data can be found in Supplementary Table S1. The TSS-seq data for human TSSs are available in the DBTSS (46). The human *trans*-splice acceptor site data are derived from published papers (10,13,29,30). Human cap-analysis of gene expression (CAGE) data are available in the ZENBU (47).
