# Supplementary material for: Characterization of trans-spliced chimeric RNAs: insights into the mechanism of trans-splicing
Source: NAR Genom Bioinform. 2024 Jun 6;6(2):lqae067. doi: 10.1093/nargab/lqae067 (PMC11155486; doi:10.1093/nargab/lqae067)
Supplement: lqae067_Supplemental_Files [file lqae067_supplemental_files.zip › supplemental_figures.docx]

Figure S1: Novel transcripts for troponin I gene. (A) Novel transcripts and the annotated KY2019 (1) transcripts for troponin I gene were shown in pink and blue, respectively. (B) Red, blue, and green points represent TSS, TAS, and the donor site, respectively. (C, D) The distribution of TSSs and TASs in each sample was shown in C and D, respectively. The x-axis and y-axis represent the position and the number of reads, respectively. BWM, body wall muscle; NC, neural complex

Figure S2: Bar plot showing the distribution of *trans*-spliced genes based on the number of TSS-seq-based *trans*-splice acceptor sites. The number in parenthesis represents the gene count.

Figure S3: Histogram showing the distances of minor sites relative to major sites. Distances between minor and major site pairs (n=24,184) were calculated. The histogram was plotted using a bin width of 5 bp. The distances within 200 bp of major sites are shown. Negative distances indicate minor sites located upstream of major sites, while positive distances indicate minor sites downstream of major sites.

Figure S4: Bar plots showing the proportion of *cis*-splice acceptor sites relative to the total number of *cis*-splice acceptor sites. In *Ciona*, the major *trans*-splice acceptor sites of 1,252 *trans*-spliced genes were located at their *cis*-spliced acceptor sites. The red bars represent the proportion of *cis*-spliced acceptor sites relative to the total number of *cis*-spliced acceptor sites that underwent *trans*-splicing. The x-axis represents the first, the second, and the third and subsequent *cis*-splice acceptor sites. The number in parenthesis indicates the number of *cis*-splice acceptor sites. The green bars represent the proportion of *cis*-spliced acceptor sites relative to the total number of *cis*-spliced acceptor sites from all transcripts of the 1,252 genes. These proportions were considered as background proportions when assuming that *trans*-splicing occurs randomly at *cis*-splice acceptor sites of these genes. In humans, 48 *trans*-splice acceptor sites from 49 *trans*-spliced genes were located at their *cis*-spliced acceptor sites. The green bars represent the proportion of *cis*-spliced acceptor sites relative to the total number of *cis*-spliced acceptor sites from all transcripts of the 49 genes. A two-sided binomial test was performed to test whether the proportion of the first *cis*-spliced acceptor sites that underwent *trans*-splicing was significantly different from the background proportion. *P*-values were less than 2.2e-16 in both *Ciona* and humans.

Figure S5: Histograms showing the distribution of distances (in base pairs) between identified TSSs and the nearest annotated TSSs in KY2019 (1). The histograms were plotted using a bin width of 30 bp. The 5′ end positions of non-SL type transcripts in KY2019 were defined as annotated TSSs. Genes lacking annotated TSSs were excluded from the histograms. Negative distances represent that the identified TSSs were located upstream of the nearest annotated TSSs, while positive distances represent that they were located downstream of the nearest annotated TSSs.

Figure S6: Violin plots showing the splice site scores of TS^High^ and TS^Low^ genes. Outliers, which fall below the first quartile − 1.5 × interquartile range (IQR) or above the third quartile + 1.5 × IQR were removed. Two-sided Mann-Whitney U test was used for statistical comparison. *P*-values from the 1st DS and AS score comparisons were adjusted using FDR correction (the Benjamini-Hochberg procedure) (2). FDRs were shown on the violin plots. TSR, *trans*-splicing ratio; DS, donor site; AS, acceptor site.

Figure S7: Histograms showing the distribution of sequence lengths for the 1st, 2nd, 3rd, and 4th exons and introns (EI) in *Ciona* (A) and humans (B). The histograms were plotted using a bin size of 20 bp. The total number of sequences (n), average length (avg.), and median length (med.) were displayed. Only sequences with lengths less than 1500 bp were shown in the histograms.

Figure S8: Nucleotide content analysis. Violin plots showing the N content (A) and N+N content (B) of the 1st, 2nd, 3rd, and 4th exons and introns in *trans*-spliced (TS-1stAS) and non-*trans*-spliced (non-TS) genes. Two-sided Mann-Whitney U test was performed for each nucleotide and feature (the 1st, 2nd, 3rd, or 4th exon and intron). *P*-values from 16 (A) or 24 (B) comparisons were adjusted using FDR correction (the Benjamini-Hochberg procedure) (2). FDRs were shown on the violin plots. ***, FDR < 0.001; **, FDR < 0.01; *, FDR < 0.05.

Figure S9: Line plots showing the median of nucleotide content in N content (A) and N+N content (B). Asterisks represent FDRs shown in Figure S8. ***, FDR < 0.001; **, FDR < 0.01; *, FDR < 0.05.

Figure S10: Line plots showing the pattern of log2 fold change (log2FC) of the median (TS-1stAS vs non-TS) in N content (A) and N+N content (B).

Figure S11: Local enrichment of (A) N content and (B) N+N content in 5′ regions. *Trans*-spliced genes were classified into four groups according to their outron length: 1) less than 150 bp, 2) 150 bp or more and less than 300 bp, 3) 300 bp or more and less than 500 bp, and 4) 500 bp or more. The line graph in each panel shows nucleotide content calculated using a 30-bp sliding window. The histogram on the top shows the frequency of *trans*-splice acceptor sites (TASs) of *trans*-spliced genes. The heatmap on the bottom shows the fold enrichment (FE) of nucleotide content in the window of *trans*-spliced genes relative to non-*trans*-spliced (non-TS) genes. Black points on the heatmap represent the statistical significance (FDR) level of each window. Background sequences (BG) were generated by randomly extracting sequences from intergenic regions. The number of genes or sequences in each group was shown in parentheses.

Figure S12: Local enrichment analysis of (A) N content and (B) N+N content around the first acceptor sites (-300 bp to +150 bp relative to the first splice sites). The line graph shows nucleotide content calculated using a 30-bp sliding window. The red and blue histograms on the top show the frequency of minor *trans*-splice acceptor sites (TASs) in TS-1stAS and TS-5UTR genes, respectively, with the density plot of the minor TAS frequency overlaying the histograms. The heatmap on the bottom shows the fold enrichment (FE) of nucleotide content in the window of TS-1stAS or TS-5UTR genes relative to non-*trans*-spliced (non-TS) genes. Black points on the heatmap represent the statistical significance (FDR) level of enrichment in each window. Background sequences (BG) were generated by randomly extracting sequences from intergenic regions. The number of genes in each group was shown in parentheses.

Figure S13: Local enrichment of (A) N content and (B) N+N content around the first acceptor sites. *Trans*-spliced genes were classified into four groups according to their outron length: 1) less than 150 bp, 2) 150 bp or more and less than 300 bp, 3) 300 bp or more and less than 500 bp, and 4) 500 bp or more. The line graph shows nucleotide content calculated using a 30-bp sliding window. The red and blue histograms on the top show the frequency of minor *trans*-splice acceptor sites (TASs) in TS-1stAS and TS-5UTR genes, respectively, with the density plot of the minor TAS frequency overlaying the histograms. The heatmap on the bottom shows the fold enrichment (FE) of nucleotide content in the window of TS-1stAS or TS-5UTR genes relative to non-*trans*-spliced genes. Black points on the heatmap represent the statistical significance (FDR) level of enrichment in each window. Background sequences (BG) were generated by randomly extracting sequences from intergenic regions. The number of genes in each group was shown in parentheses.

Figure S14: The nucleotide sequence of *Ciona* SL RNA. *Ciona* SL RNA has a SL exon, a *trans*-splice donor site, and a predicted Sm protein binding site (3). The complementary sequence of the 3′region has AU- and GU-rich sites.

References

1. Satou, Y., Nakamura, R., Yu, D., Yoshida, R., Hamada, M., Fujie, M., Hisata, K., Takeda, H. and Satoh, N. (2019) A Nearly Complete Genome of Ciona intestinalis Type A (C. robusta) Reveals the Contribution of Inversion to Chromosomal Evolution in the Genus Ciona. *Genome Biol Evol*, **11**, 3144-3157.

2. Benjamini, Y. and Hochberg, Y. (1995) Controlling the False Discovery Rate: A Practical and Powerful Approach to Multiple Testing. *Journal of the Royal Statistical Society: Series B (Methodological)*, **57**, 289-300.

3. Vandenberghe, A.E., Meedel, T.H. and Hastings, K.E.M. (2001) mRNA 5 '-leader trans-splicing in the chordates. *Genes & Development*, **15**, 294-303.
