# Supplementary material for: Characterization of trans-spliced chimeric RNAs: insights into the mechanism of trans-splicing
Source: NAR Genom Bioinform. 2024 Jun 6;6(2):lqae067. doi: 10.1093/nargab/lqae067 (PMC11155486; doi:10.1093/nargab/lqae067)
Supplement: lqae067_Supplemental_Files [file lqae067_supplemental_files.zip › supplemental_methods.docx]

# Supplemental Methods

# Re-assembling transcripts

# RNA-seq data of *Ciona intestinalis* type A (*Ciona robusta*)

A total of 82 RNA-seq samples (Table S1) were obtained from the sequence read archive (SRA) database (https://www.ncbi.nlm.nih.gov/sra).

# Preprocessing and filtering of RNA-seq reads

Low quality 5′ and 3′ ends of reads were trimmed using cutadapt v1.11 (Martin 2011) with the options –q 10 –e 0.1. Adapters were trimmed using cutadapt v1.11 with the option –e 0.1 (Table S2). The 16-nt spliced-leader (SL) sequence was trimmed using cutadapt v1.11 with the option –e 0.1. Poly(A) sequences were trimmed using cutadapt v1.11 with the options -a A -g T. The reads of less than 36 nt length were removed. The survived reads were mapped to rRNA sequences using bowtie v2.2.6 (Langmead et al. 2009) with default settings. The rRNA sequences including *Ciona* rRNAs were obtained from the SILVA database (SSU Ref Nr99 and LSU Ref release 128) (Quast et al. 2013) (https://www.arb-silva.de) and the NCBI database (https://www.ncbi.nlm.nih.gov). The reads mapped to rRNAs were removed.

# Mapping RNA-seq reads

The preprocessed and filtered reads were mapped to the reference genome, the HT (Hoya T-strain) genome (primary chromosomes only) (http://ghost.zool.kyoto-u.ac.jp/download_ht.html), using STAR v2.7.9a (Dobin et al. 2013) with the help of the gene annotation (KY2019 gene model; core models only) and the following options: --twopassMode Basic --outSAMstrandField intronMotif --outFilterIntronMotifs RemoveNoncanonicalUnannotated --outSJfilterOverhangMin 1 1 1 1 --outSJfilterCountUniqueMin 0 0 0 0 --outSJfilterCountTotalMin 1 1 1 1 --outSJfilterDistToOtherSJmin 0 0 0 0 --outSJfilterIntronMaxVsReadN 0 --alignSoftClipAtReferenceEnds No --alignIntronMax 46000.

# Assembling transcripts

Transcripts were assembled for each sample using StringTie v1.2.3 (Pertea et al. 2015) and Scallop v0.10.4 (Shao and Kingsford 2017) with and without the gene annotation. The assembled transcripts from StringTie and Scallop were merged into a non-redundant set of transcripts using StringTie merge and TACO v0.7.3 (Niknafs et al.), respectively.

# Identifying novel transcripts with outrons

The non-redundant transcripts were split at known operon *trans*-splice acceptor sites. Transcripts whose exons overlapped multiple genes were removed. The coding region (CDS) of each transcript was estimated based on the longest open-reading frame (ORF). Transcripts that did not have ORFs were removed. Known transcript IDs were assigned to assembled transcripts based on the overlap of the CDS and/or splice sites. For each known SL and ND transcript in the KY model, transcripts with novel 5′ exons or extended 5′ ends were considered novel transcripts with outrons.

# Identification of *trans*-spiced genes

# Identifying *Ciona* *trans*-splice acceptor sites

TSS-seq reads were preprocessed as described in the previous study (Yokomori et al. 2016). The preprocessed reads containing the spliced leader (SL) sequence at their 5′ end (referred to as SL+ reads) were extracted. The SL sequences were then removed from the SL+ reads. The SL+ reads were mapped to the HT genome (primary chromosomes only) using STAR v2.7.9a (Dobin et al. 2013) with the help of the gene annotation (KY2019 gene model; core models only) and the following options: --outWigType wiggle read1_5p --outWigStrand Stranded --outWigNorm None --twopassMode Basic --alignEndsType Extend5pOfRead1. Uniquely mapped reads were used to identify *trans*-splice acceptor sties. The following positions were considered TSS-seq-based *trans*-splice acceptor sites: 1) the 5′ end positions of the reads mapped to annotated *trans*-splice acceptor sites (i.e. the 5′ ends of SL-type transcripts) and 2) the 5′ end positions of the mapped reads with the AG motif immediately upstream of them.

# Identifying human *trans*-splice acceptor sites

Human *trans*-splice acceptor sites were retrieved from a published paper (Babiceanu et al. 2016), which provided 291 recurrent chimeric fusion RNAs detected in more than one sample. In the published paper, they were classified into three classes based on the genomic location of their parental genes: 1) fusions whose parental genes are neighboring genes and transcribed on the same strand, 2) fusions whose parental genes are located on different chromosomes, 3) other fusions whose parental genes are located on the same chromosome. Out of the three classes, fusion RNAs of the latter two classes were considered putative *trans*-spliced RNAs. The first class was ignored in this study because they are thought to be produced by *cis*-splicing between adjacent genes (Jia et al. 2016). The junction positions of parental upstream genes and downstream genes were regarded as *trans*-spliced donor sites (TDSs) and *trans*-splice acceptor sites (TASs), respectively. Moreover, to obtain more reliable TDS-TAS pairs, those with GU-AG splice site motifs were selected. We also added TDS-TAS pairs of six experimentally validated *trans*-spliced chimeric RNAs from other published papers, all of which have GU-AG splice site motifs (Li et al. 2008; Yuan et al. 2013; Wu et al. 2014). The coordinates of all the sites were based on the GRCh37/hg19 assembly.

# Classifying *Ciona* genes into *trans*-spliced and non-*trans*-spliced genes

*Ciona* genes in the KY2019 core model were first classified into operonic and non-operonic genes. Operons are polycistronic regions where two or more genes are present consecutively on the same strand with no intergenic distance, and where the 5' ends of transcripts from all the genes except the first gene are *trans*-splice acceptor sites. Non-operonic genes were subsequently classified into *trans*-spliced and non-*trans*-spliced genes using annotated *trans*-splice acceptor sites in the KY2019 model and TSS-seq-based *trans*-splice acceptor sites. *Trans*-spliced genes were defined as genes that have TSS-seq-based *trans*-splice acceptor sites on their gene body. If they do not have TSS-seq-based *trans*-splice acceptor sites, but have annotated *trans*-splice acceptor sites, they were considered ambiguous genes and not used in this study. The remaining genes were classified into *trans*-spliced genes.

# Location of *trans*-splice acceptor and donor sites

*Trans*-splice acceptor site locations were placed into the following categories: 1) acceptor site (AS) , 2) intron, 3) 5′ UTR, 4) CDS, 5) 3′ UTR . If the *trans*-splice acceptor site can be placed into multiple categories due to overlapping transcripts, its location was determined according to the following priority: 1) AS, 2) intron, 3) 5′ UTR, 4) CDS, 5) 3′ UTR.

# Estimation of *RMST* transcripts

The RNA-seq data of human ESCs (SRR5444924) was obtained from the SRA database (https://www.ncbi.nlm.nih.gov/sra). TruSeq paired-end adapters were trimmed using cutadapt v1.11 with options: -q 10,10 -e 0.1 -m 36 (Martin 2011). The trimmed reads were mapped to the hg19 genome using STAR v2.5.3a (Dobin et al. 2013). Transcripts were then estimated using StringTie v1.2.3 (Pertea et al. 2015) with the RefSeq gene annotation.

# Identification of transcription start sites (TSSs) for *Ciona* genes

# Identifying open chromatin regions

ATAC-seq reads (Racioppi et al. 2019) were preprocessed using fastp v0.20.0 (Chen et al. 2018). The preprocessed reads were mapped to the HT gnome (the 14 chromosomes only) using bowtie v2.4.1 with the options -N 1 -X 2000 (Langmead et al. 2009). Duplicated reads were removed using GATK v4.1.4.1 MarkDuplicates. Only uniquely mapped reads were used for peak calling. ATAC-seq peaks and their summits were called using MACS v2.2.7.1 with the options --format BAMPE --keep-dup all --gsize 1.23e8 --call-summits --control <gDNA.bam> (Zhang et al. 2008). To detect the most accessible open chromatin regions, the high density regions of peak summits were estimated using F-Seq (Boyle et al. 2008).

# Identifying TSSs

TSS-seq reads (Yokomori et al. 2016) were mapped to the HT genome (the 14 chromosomes only) using STAR v2.7.9a (Dobin et al. 2013). TSS clusters (TSCs) were identified using a sliding window method (Yokomori et al. 2016). Next, we identified the TSCs associated with each transcript for every gene group as described below. For TS-5UTR genes, we identified TSCs within the region upstream of the *trans*-splice acceptor site of the transcript. For TS-1stAS genes, we identified TSCs within the region from 200 bp upstream of the 5′ end of the transcript to the first donor site. For non-*trans*-spliced genes, we identified TSCs within the region from 200 bp upstream of the 5' end of the transcript to either the first donor site or the translation start site, whichever was further upstream. Then we selected only the TSCs overlapping with open chromatin regions (ATAC-seq high density regions) from those identified in each transcript. If multiple TSCs were selected, the one nearest to the highest density signal value in the high density regions was selected as the representative one. The putative TSS was determined based on the most frequent position in the representative TSC. To improve data quality, we excluded transcripts meeting specific criteria. Transcripts of TS-5UTR genes were excluded if they had very short outrons of less than 51bp, if they had splice donor sites between their TSS and *trans*-splice acceptor site, or if the TSC overlapped with the *trans*-splice acceptor site. Transcripts of TS-1stAS genes were excluded if they had very short first exon of less than 4 bp or if the TSC overlapped with the *trans*-splice acceptor site. Transcripts of non-*trans*-spliced genes were excluded if the number of TSS-seq reads in the TSC was small (< 30) or if there were any *trans*-splice acceptor sites between the TSS and 3′ end. The genes in each group were excluded if no transcripts were retained. Additionally, for each gene group, we excluded genes with multiple transcripts having different TSCs (i.e. alternative promoters) to simplify the analysis.

# Motif Analysis

# Human RNA-binding protein motifs

The motifs of human RNA-binding proteins (RBPs) were retrieved from the ATtRACT database (version 0.99b) (https://attract.cnic.es) (Giudice et al. 2016). The motifs with score “1” and/or made from only one binding site sequence were removed. A total of 12 motifs of six different human hnRNPs were obtained from a previous paper (Huelga et al. 2012) and added into a set of human RBP motifs. The motif redundancy in the human RBP motif set was removed to obtain a non-redundant set.

# Removal of motif redundancy

The similarity between motif pairs was estimated using TOMTOM version 5.0.1 (Gupta et al. 2007) with the options “-norc -thresh 1”. The edges of motifs with low information content less than 0.4 were trimmed before running TOMTOM to assist accurate search. The motifs were then clustered using complete linkage hierarchical clustering to obtain motif clusters, in each of which motifs are significantly similar to each other. The *q*-values from TOMTOM were used as distances between motif pairs. The clustering was repeated until the maximum distance (*q*-value) between motif pairs in a cluster exceeds 0.01. The motifs in each cluster were aligned using STAMP version 1.3 (Mahony and Benos 2007) with the options “-cc PCC -align SWU -forwardonly”. The aligned motifs were merged to obtain a non-redundant set of motifs. The edges of merged motifs with information content less than 0.4 were trimmed. The merged motifs of less than 4-bp length were discarded after edge trimming.

# Prediction of motif binding sites

Motif binding sites were predicted using FIMO version 5.0.1 (Grant et al. 2011) with the *P*-value threshold 0.001. The 3rd order Markov model of intergenic sequences of the reference genome was used as a background model. The 5′ end position of the predicted site was used as the motif position.

# Identification of homologs

*Ciona* protein sequences (KY2019) were aligned to RefSeq human protein sequence database using BLAST+ version 2.10.1(Camacho et al. 2009).

References

Babiceanu M, Qin F, Xie Z, Jia Y, Lopez K, Janus N, Facemire L, Kumar S, Pang Y, Qi Y et al. 2016. Recurrent chimeric fusion RNAs in non-cancer tissues and cells. *Nucleic Acids Res* **44**: 2859-2872.

Boyle AP, Guinney J, Crawford GE, Furey TS. 2008. F-Seq: a feature density estimator for high-throughput sequence tags. *Bioinformatics* **24**: 2537-2538.

Camacho C, Coulouris G, Avagyan V, Ma N, Papadopoulos J, Bealer K, Madden TL. 2009. BLAST+: architecture and applications. *BMC Bioinformatics* **10**: 421.

Chen S, Zhou Y, Chen Y, Gu J. 2018. fastp: an ultra-fast all-in-one FASTQ preprocessor. *Bioinformatics* **34**: i884-i890.

Dobin A, Davis CA, Schlesinger F, Drenkow J, Zaleski C, Jha S, Batut P, Chaisson M, Gingeras TR. 2013. STAR: ultrafast universal RNA-seq aligner. *Bioinformatics* **29**: 15-21.

Giudice G, Sánchez-Cabo F, Torroja C, Lara-Pezzi E. 2016. ATtRACT-a database of RNA-binding proteins and associated motifs. *Database (Oxford)* **2016**.

Grant CE, Bailey TL, Noble WS. 2011. FIMO: scanning for occurrences of a given motif. *Bioinformatics* **27**: 1017-1018.

Gupta S, Stamatoyannopoulos JA, Bailey TL, Noble WS. 2007. Quantifying similarity between motifs. *Genome Biol* **8**: R24.

Huelga SC, Vu AQ, Arnold JD, Liang TY, Liu PP, Yan BY, Donohue JP, Shiue L, Hoon S, Brenner S et al. 2012. Integrative genome-wide analysis reveals cooperative regulation of alternative splicing by hnRNP proteins. *Cell Rep* **1**: 167-178.

Jia Y, Xie Z, Li H. 2016. Intergenically Spliced Chimeric RNAs in Cancer. *Trends in Cancer* **2**: 475-484.

Langmead B, Trapnell C, Pop M, Salzberg SL. 2009. Ultrafast and memory-efficient alignment of short DNA sequences to the human genome. *Genome Biol* **10**: R25.

Li H, Wang J, Mor G, Sklar J. 2008. A neoplastic gene fusion mimics trans-splicing of RNAs in normal human cells. *Science* **321**: 1357-1361.

Mahony S, Benos PV. 2007. STAMP: a web tool for exploring DNA-binding motif similarities. *Nucleic Acids Res* **35**: W253-258.

Martin M. 2011. Cutadapt removes adapter sequences from high-throughput sequencing reads. *EMBnetjournal; Vol 17, No 1: Next Generation Sequencing Data Analysis*.

Niknafs YS, Pandian B, Iyer HK, Chinnaiyan AM, Iyer MK. TACO produces robust multisample transcriptome assemblies from RNA-seq.

Pertea M, Pertea GM, Antonescu CM, Chang TC, Mendell JT, Salzberg SL. 2015. StringTie enables improved reconstruction of a transcriptome from RNA-seq reads. *Nat Biotechnol* **33**: 290-295.

Quast C, Pruesse E, Yilmaz P, Gerken J, Schweer T, Yarza P, Peplies J, Glöckner FO. 2013. The SILVA ribosomal RNA gene database project: improved data processing and web-based tools. *Nucleic Acids Res* **41**: D590-596.

Racioppi C, Wiechecki KA, Christiaen L. 2019. Combinatorial chromatin dynamics foster accurate cardiopharyngeal fate choices. *Elife* **8**.

Shao M, Kingsford C. 2017. Accurate assembly of transcripts through phase-preserving graph decomposition. *Nat Biotechnol* **35**: 1167-1169.

Wu CS, Yu CY, Chuang CY, Hsiao M, Kao CF, Kuo HC, Chuang TJ. 2014. Integrative transcriptome sequencing identifies trans-splicing events with important roles in human embryonic stem cell pluripotency. *Genome Res* **24**: 25-36.

Yokomori R, Shimai K, Nishitsuji K, Suzuki Y, Kusakabe TG, Nakai K. 2016. Genome-wide identification and characterization of transcription start sites and promoters in the tunicate Ciona intestinalis. *Genome Res* **26**: 140-150.

Yuan H, Qin F, Movassagh M, Park H, Golden W, Xie Z, Zhang P, Sklar J, Li H. 2013. A chimeric RNA characteristic of rhabdomyosarcoma in normal myogenesis process. *Cancer Discov* **3**: 1394-1403.

Zhang Y, Liu T, Meyer CA, Eeckhoute J, Johnson DS, Bernstein BE, Nusbaum C, Myers RM, Brown M, Li W et al. 2008. Model-based analysis of ChIP-Seq (MACS). *Genome Biol* **9**: R137.
